# Interactions of tumour-derived micro(nano)vesicles with human gastric cancer cells

**DOI:** 10.1186/s12967-015-0737-0

**Published:** 2015-12-01

**Authors:** Małgorzata Stec, Rafał Szatanek, Monika Baj-Krzyworzeka, Jarosław Baran, Maria Zembala, Jakub Barbasz, Agnieszka Waligórska, Jurek W. Dobrucki, Bożenna Mytar, Antoni Szczepanik, Maciej Siedlar, Grażyna Drabik, Barbara Urbanowicz, Marek Zembala

**Affiliations:** Department of Clinical Immunology and Transplantology, Jagiellonian University Medical College, Wielicka 265 Str., 30-663 Kraków, Poland; Institute of Catalysis and Surface Chemistry, Polish Academy of Sciences, Kraków, Poland; Division of Cell Biophysics, Faculty of Biochemistry, Biophysics and Biotechnology, Jagiellonian University, Kraków, Poland; First Department of General and Gastrointestinal Surgery, Jagiellonian University Medical College, Kraków, Poland; Electron Microscopy Laboratory, University Children’s Hospital of Cracow, Kraków, Poland

**Keywords:** Gastric cancer, Microvesicles, NOD SCID mouse model

## Abstract

**Background:**

Tumour cells release membrane micro(nano)fragments called tumour-derived microvesicles (TMV) that are believed to play an important role in cancer progression. TMV suppress/modify antitumour response of the host, but there is also some evidence for their direct interaction with cancer cells. In cancer patients TMV are present in body fluid and tumour microenvironment. The present study aimed at characterization of whole types/subpopulations, but not only exosomes, of TMV from newly established gastric cancer cell line (called GC1415) and to define their interactions with autologous cells.

**Methods:**

TMV were isolated from cell cultures supernatants by centrifugation at 50,000×*g* and their phenotype was determined by flow cytometry. The size of TMV was analysed by dynamic light scattering and nanoparticle tracking analysis, while morphology by transmission electron microscopy and atomic force microscopy. Interactions of TMV with cancer cells were visualized using fluorescence-activated cell sorter, confocal and atomic force microscopy, biological effects by xenografts in NOD SCID mice.

**Results:**

Isolated TMV showed expression of CD44H, CD44v6 (hyaluronian receptors), CCR6 (chemokine receptor) and HER-2/neu molecules, exhibited different shapes and sizes (range 60–900 nm, highest frequency of particles with size range of 80–120 nm). TMV attached to autologous cancer cells within 2 h and then were internalized by them at 24 h. CD44H, CD44v6 and CCR6 molecules may play a role in attachment of TMV to cancer cells, while HER-2 associated with CD24 be involved in promoting cancer cells growth. Pre-exposure of cancer cells to TMV resulted in enhancement of tumour growth and cancer cell-induced angiogenesis in NOD SCID mice model.

**Conclusions:**

TMV interact directly with cancer cells serving as macro-messengers and molecular cargo transfer between gastric cancer cells resulting in enhancement of tumour growth. TMV should be considered in future as target of anticancer therapy.

## Background

Interactions/communications between tumour cells themselves and tumour cells with the microenvironment are responsible for cancer development and progression [1], but the pathomechanism(s) of cancer progression are far from clear. Tumour microenvironment plays a major role in this process, as both infiltrating and tumour cells produce a range of soluble mediators with protumour activity (e.g. interleukin-6, transforming growth factor β, epidermal growth factor and its receptor, vascular endothelial growth factor, etc.). However, immunotherapy approaches employing various monoclonal antibodies (mAbs), neutralizing the mediators or blocking their receptors, are only partially effective [2]. Therefore, there must be another mechanism(s) responsible for cancer progression.

Recently, there has been an increasing interest in studying tumour cells surface micro(nano)fragments also called micro(nano)vesicles (MV). MV are defined as spherical proteolipids with sizes ranging from 30 nm to 1 μm [3]. MV circulate in blood of healthy individuals and their elevated numbers are found in patients with various diseases, including cancer [4, 5]. They are shed by many cell types, in particular rapidly proliferating. Tumour cells, in particular more malignant, release MV called tumour-derived MV (TMV). They are present in body fluids and tumour microenvironment [1]. In some types of cancer (ovarian, breast, gastric) elevated numbers of MV, not necessarily of tumour origin, are associated with poor prognosis [4–6]. MV carry various surface determinants and mRNA/miRNA and transfer some of them to other cells (e.g. blood cells), hence are regarded as important messengers in cell to cell communication [7–9]. TMV inhibit antitumour response of the host [10] and may play a pivotal role in cancer progression [1, 11]. There is some evidence that tumour cell-derived exosomes (particles with sizes <100 nm), that TMV contain, interact with autologous tumour cells. Firstly, TMV entry to recipient cells, including prostate, lung and breast cancer [12]. Secondly, gastric cancer exosomes promote tumour cell proliferation, at least in part, by activation of PI3K/Akt and MAPK/EPK pathways [13]. Thirdly, TMV contain selectively secreted components which are required for metastasis formation, e.g. in breast and non-small lung cancer [14, 15].

We have previously described the characteristics of circulating MV in blood of gastric cancer patients, and concluded that at least some of them may be of tumour origin [5]. As little is known about TMV from gastric cancer cells, we have established the novel gastric cancer cell line (called GC1415) and characterized immunophenotype, including expression of tumour markers of cells and TMV derived from them. The aim of these studies was to characterize all particles shed/released by tumour cells, but not only isolated exosomes, i.e. particles with sizes less than 100 nm, as the latter may not be representative for whole types/subpopulations of TMV. Their size, physical properties and morphology was determined. Isolated TMV were used to define their interactions with cancer cells they derived from. The present study shows that TMV attached to the cell surface and then were internalized as visualized independently by three different methodologies. Furthermore, TMV changed some biological properties of autologous cancer cells.

## Methods

### Cancer cells

Tumour cell line GC1415 was recently established in our laboratory from carcinomatous ascites of a patient with advanced gastric (adenocarcinoma) cancer. These studies were approved by the Jagiellonian University Medical College Ethical Committee (KBET/491/B/2003). The cells were cultured in DMEM with high glucose content (PAA Laboratories GmbH, Pasching, Austria) supplemented with 5 % foetal bovine serum (FBS, Biowest, Nuaille, France) at 37 °C, in 5 % CO_2_ atmosphere and regularly tested for *Mycoplasma* sp. contamination by PCR-ELISA kit (Roche, Mannheim, Germany) and for endotoxin contamination by the Limulus test (Charles River Laboratories, Wilmington, MA) according to the manufacturers’ instruction.

### TMV isolation

GC1415 cells were cultured by bi-weekly passages in DMEM with 5 % FBS previously centrifuged at 50,000×*g* for 1 h to remove bovine MV. Supernatants from the cultures at confluency were collected and spun down at 3000×*g* for 10 min to remove cellular debris. Then, supernatants were centrifuged using RC28S ultracentrifuge (Sorvall, Newton, CT) at 50,000×*g* for 1 h at 4 °C. Pellets were washed several times in serum-free DMEM to remove FBS, and finally resuspended in it. Quantification of TMV protein concentration was evaluated by the Bradford’s method using Quick Start Bradford Dye Reagent (BioRad, Hercules, CA). The number of TMV was determined by flow cytometry (FACS Canto, BD Biosciences Immunocytometry Systems, San Jose, CA) and nanoparticle tracking analysis (NTA) using NanoSight system, i.e. LM10HS microscope equipped with the LM14 488 nm laser module (Malvern Instruments Ltd., Malvern, UK). TMV were also tested for *Mycoplasma* sp. and endotoxin contamination and stored at −20 °C for further usage.

### Flow cytometry

The following murine IgG_1_ monoclonal antibodies (mAbs) were used: phycoerythrin (PE)-conjugated anti-CD44H and fluorescein (FITC)-conjugated anti-CD44v6 (eBioscience GmbH, Vienna, Austria), PE-conjugated anti-CCR6, anti-EGFR and allophycocyanin (APC)-conjugated anti-HER-2/neu (BD Biosciences, San Diego, CA), PE-conjugated anti-Mucin1 and anti-EMMPRIN (Santa Cruz Biotechnologies, Santa Cruz, CA), FITC-conjugated anti-EpCAM (DAKO, Glostrup, Denmark). Isotype controls included appropriate FITC-, PE- or APC-labelled mouse immunoglobulins. Cancer cells (1 × 10^5^ per sample) or TMV (1 μg per sample) were incubated with appropriate saturating concentrations of mAbs for 20 min at 4 °C, washed (except TMV), resuspended in phosphate buffered saline (PBS) and analysed by FACS.

### Determination of HER-2/neu and MAGE-1, -2 mRNA expression

Total RNA was isolated from the GC1415 tumour cells (1 × 10^6^) and their TMV using the RNeasy Mini Kit (Qiagen, Hilden, Germany) according to the manufacturer’s protocol. The cDNA synthesis and nested, “real-time” PCR for MAGE-1, -2 and β-actin was performed as previously described [16]. For the detection of HER-2/neu mRNA “real time” PCR was performed using the following primer pair: for HER-2/neu: sense-5′-CCTCTGACGTCCATCATCTC-3′ and antisense-5′-ATCTTCTCGTGCCGTCGCTT-3′. The cycle profile for HER-2/neu PCR run was as follows: initial denaturation at 95 °C for 10 min, then denaturation at 95 °C for 0 s, annealing at 60 °C for 35 s, and elongation at 72 °C for 35 s for 35 cycles, followed by final extension at 72 °C for 2 min. All the results were normalized with β-actin data and were presented as ΔC_T_. To verify the amplified product, melting curve analysis using the 2.3 LightCycler software was performed for each sample.

### Determination of TMV size distribution

Size distribution using the dynamic light scattering (DLS) was determined as previously described [5]. Briefly, samples either diluted or not (depending on signal intensity) in 0.15 M NaCl were measured in Zetasizer Nano ZS apparatus (Malvern Instruments) equipped with laser of λ = 633 nm. Size distribution was obtained from measured diffusion coefficients recalculated to the size by assuming spherical shape of particles. The obtained values represent the diameter of spherical particles which move in viscous media with the same velocity as TMV. The size and distribution of TMV was also determined by NTA. For this purpose TMV samples were diluted 200 times in prefiltered (0.2 μm), sterile PBS to the total volume of 1 ml, then aspirated into an insulin-type syringe and loaded into the sample chamber. The TMV size and distribution measurements were collected in triplicates of 1 min film intervals recorded by the sCMOS camera and calculated using the NanoSight NTA 2.3 analytical software.

### Zeta potential determination

Particle electrophoretic mobility was measured with Zetasizer Nano ZS apparatus (Malvern Instruments) in samples 10 times diluted with distilled water (to final ionic strength equal to 0.015 M) and recalculated to Zeta potential using Smoluchowsky’s equation, as previously described [6].

### Transmission electron microscopy (TEM)

Purified TMV were mixed with 2 % of uranyl acetate and applied to carbon coated copper grid. The microphotographs were taken with Philips EM 300 electron microscope (Philips, Eindhoven, The Netherlands) operating at 80 kV.

### Atomic force microscopy (AFM)

AFM was used to determine morphology of TMV. Samples for AFM were prepared from TMV suspensions by adsorbing onto highly ordered pyrolytic graphite (ZYH—mosaic spread 3.5–5.0) supports (NT-MDT Co., Zelenograd, Russia). The measurements were performed using Ntegra Vita microscope (NT-MDT Co.). The AFM images were recorded in semicontact mode. Experiments were performed using the commercial silicon tip with polysilicon cantilever (NT-MDT Co.) and resonance frequency within the range of 140–240 kHz and spring constants k = 3.4 N/m or k = 5.8 N/m. AFM imaging of cancer cells were acquired in phase contact mode, which reflects both morphology and mechanical properties of the sample. Image Analysis 2.2.0 software from NT-MDT Co. was used for AFM image processing.

### Binding and engulfment of TMV

#### FACS analysis

To test the ability of TMV to bind to tumour cells, TMV were stained with red PKH26 fluorescent dye (Sigma-Aldrich, St. Louis, MO) for 5 min, according to the manufacturer’s instruction and then incubated with GC1415 cells (app. 10 TMV per cell) for 2 and 24 h. To discriminate between the cells which already engulfed TMV (intracellular localization) from those with TMV only attached to their surface (extracellular) quenching assay with crystal violet (Sigma-Aldrich, 1 mg/ml final concentration), was applied according to the manufacturer’s instructions [17]. The samples were then analysed by FACS, where gate P1 was set according to autofluorescence of GC1415 cells incubated in the medium without TMV (negative control).

#### AFM analysis

For AFM analysis, tumour cells (2 × 10^4^ per well) were cultured in DMEM with 1 % FBS for 48 h on Lab-Tek Chamber Slide (Nalgene Nunc International, Naperville, IL). Then, cells were incubated with TMV (at the ratio 1:10) for the next 24 h, washed and assessed by AFM, as described above, except that the cells were analysed directly on the slide and pictures were presented in phase contrast mode, which reflects both morphology and mechanical properties of the sample.

#### Confocal microscopy analysis

GC1415 cells (1 × 10^6^ per ml) were cultured in DMEM with 10 % FBS on Super Frost Plus microscopy slides (Menzel-Glaser, Braunschweig, Germany) for 48 h. TMV were incubated for 5 min with red PKH26 dye (Sigma-Aldrich) according to manufacturer’s instruction. Then, TMV were washed with 1 % bovine serum albumin and several times in serum-free DMEM medium. PKH26-labelled TMV resuspended in the medium were added to GC1415 cells and incubated for 2 and 24 h. As control the cells were processed identically without TMV. After incubation, cells with intact plasma membrane were labelled with fluorescein, following hydrolysis of fluorescein diacetate (Sigma-Aldrich), as described by Rotman and Papermaster [18]. Life cell imaging was used to visualize extra- and intracellular localization of TMV (red fluorescence, GC1415 cells-green fluorescence). Images were acquired by Leica TCS SP5 laser scanning confocal microscope (Leica Microsystems CMS GmbH, Mannheim, Germany) equipped with 63x, NA1.4 planapochromatic oil immersion objective. During imaging cells were maintained at 37 °C in DMEM/F12 medium without phenol red (Sigma-Aldrich), supplemented with 2 % FBS. The confocal pinhole size was set at 1 Airy unit. PKH26 fluorescence was excited with 543 nm line of He/Ne diode laser and emission was recorded in the range of 570–640 nm. For excitation of fluorescein 488 nm line of argon ion laser was used and the emission was recorded in the range of 500–540 nm. Image processing and analyses were performed by LAS AF (Leica Microsystems) software.

#### Xenografts of GC1415 cells in NOD SCID mice

The GC1415 cells (1 × 10^6^ per mouse) suspended in 200 μl of PBS were injected subcutaneously into dorsal region of 8-weeks old NOD SCID mice purchased from Charles River Laboratories (Sulzfeld, Germany). Handling of mice and all procedures were performed under laminar flow conditions. In some experiments tumour cells were preincubated with autologous TMV (9 × 10^6^) for 2 or 24 h, washed and then injected. The cells preincubated in the culture medium alone were used as control. Mice (5 per group) were checked every 7 days and the tumour diameter was measured with a caliper and its volume (v) was calculated according to the formula: v = ab^2^/2 [19]. The tissues were examined macroscopically for metastases in the spleen, liver, lung, lymph nodes, kidney and then processed for histological examination.

#### Angiogenesis assay

Tumour cells, with or without TMV, were prepared as above, washed and resuspended in 100 μl of PBS. Then, cells were mixed with 400 μl of Matrigel Matrix (BD Biosciences) and injected subcutaneously into the abdominal middle line of NOD SCID mice. After 6 days mice were euthanized, Matrigel Matrix pellets were cut out and then 400 μl of Cell Recovery Solution (BD Biosciences) was added and left at 4 °C for 7 days. Then, Matrigel Matrix implants were suspended in Drabkin’s solution (Sigma-Aldrich) for 30 min, after that spun down and the content of haemoglobin was determined by measuring the absorbance (O.D.) at 540 nm in U-1800 spectrophotometer (Hitachi, Tokyo, Japan) [20]. Independently Matrigel plugs after cutting out were photographed and then fixed in formalin, paraffin embedded, serial 3 μm sections were cut and stained with hematoxylin and eosin.

### Statistical analysis

The nonparametric Mann–Whitney two-sided test was performed using GraphPad InStat version 2.0 software. *P* values <0.05 were considered significant.

## Results

### Immunophenotype of tumour cells and TMV

**Fig. 1:**
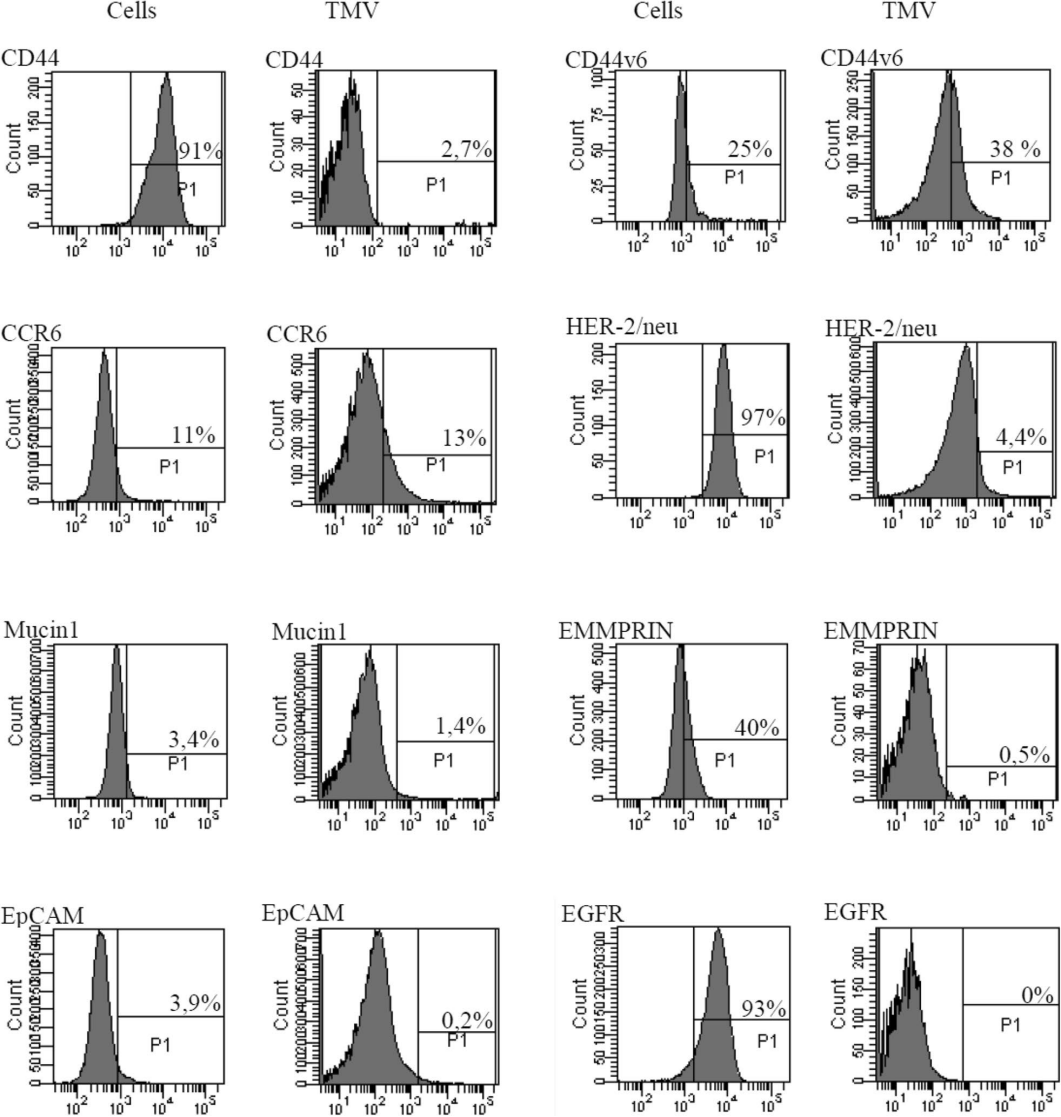
Expression of some CD determinants and tumour markers on GC1415 cells and their TMV. Immunophenotype was determined by FACS analysis

The CD44H (hyaluronian receptors), CD44v6, CCR6 (chemokine receptors) and EGFR (epithelial growth factor receptor) are involved in interactions of tumour cells with monocytes [21, 22]. The question was raised whether these receptors, by analogy, play a similar role in TMV-cancer cells interactions. Therefore, appropriate mAbs against these determinants were used to detect their expression on GC1415 cells and TMV. The presence of some tumour markers was also determined. The comparison of expression of tested determinants showed remarkable differences between tumour cells and their TMV (Fig. 1). The majority of cells were positive for CD44H and EGFR and about 50 % for CD44v6. The expression of EMMPRIN was observed on app. 20–60 % cells, CCR6 on 10–14 %, while few cells were EpCAM, EGFR and Mucin1 positive. Among analysed tumour markers, HER-2/neu was detected on all cells, but CEA and TAG-72 presence was not observed. The expression level of CD44H, EMMPRIN, HER-2/neu, EpCAM, EGFR and Mucin1 on TMV was significantly lower or absent, except CD44v6 and CCR6 which was comparable on cells and their TMV. The staining for MAGE-1, -2 was not performed due to the lack of suitable mAbs.

### The MAGE-1, -2 and HER-2/neu mRNA expression

The expression of mRNA for MAGE-1, 2 and HER-2/neu was detected in both the cells and TMV, however, it was lower in TMV compared to the GC1415 tumour cells as viewed by the threshold cycle value (ΔC_T_). For the mRNA expression of MAGE-1, -2 and HER-2/neu in TMV the ΔC_T_ values were as follows: 6.163 ± 1.439, 7.897 ± 1.385, 6.890 ± 1.473, where as in the GC1415 tumour cells they were 4.990 ± 3.167, 2.673 ± 4.031, 5.21 ± 2.598, respectively. Melting curve analysis confirmed the specificity of the PCR products.

### The physical characteristics of TMV

**Fig. 2:**
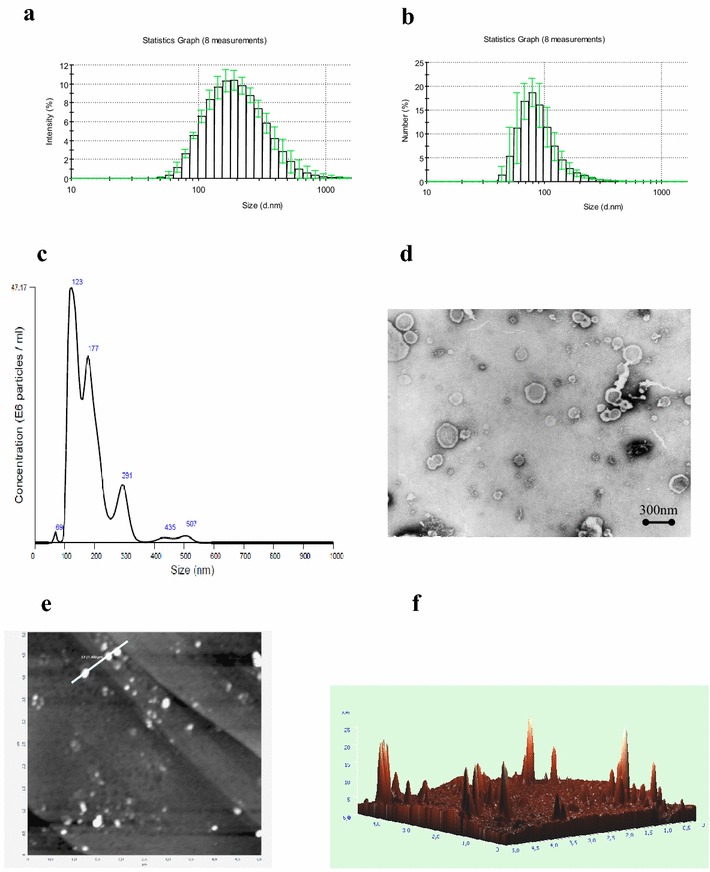
The physical characteristics of TMV determined by DLS, NTA, TEM and AFM. **a** Size range and **b** size frequency of TMV by DLS. **c** Representative size frequency by NTA. **d** TEM, **e** 2D AFM and **f** 3D images (5 × 5 µm area) of TMV deposited onto pyrolytic graphite. Sizing of objects present along the *line* marked in part (**e**): *x*-axis—size, *y*-axis—height

The effective size range of TMV as determined by DLS analysis was between 60–900 nm (Fig. 2a) and the most frequent events were around 80 nm (Fig. 2b). By NTA the reciprocal values were 70–520 nm and around 120 nm (Fig. 2c), respectively. Electrokinetic (Zeta) potential of TMV, which define the effective charge of objects, was −23.5 ± 1.80 mV. TEM and AFM were used to determine the morphology and size of TMV. TMV did not exhibit any characteristic features and were of different sizes and shapes, mostly spheroid (Fig. 2d, e). Base cross-sections of selected particles (Fig. 2f) were within size limits, determined by DLS.

### Binding and engulfment of TMV by autologous cancer cells

**Fig. 3:**
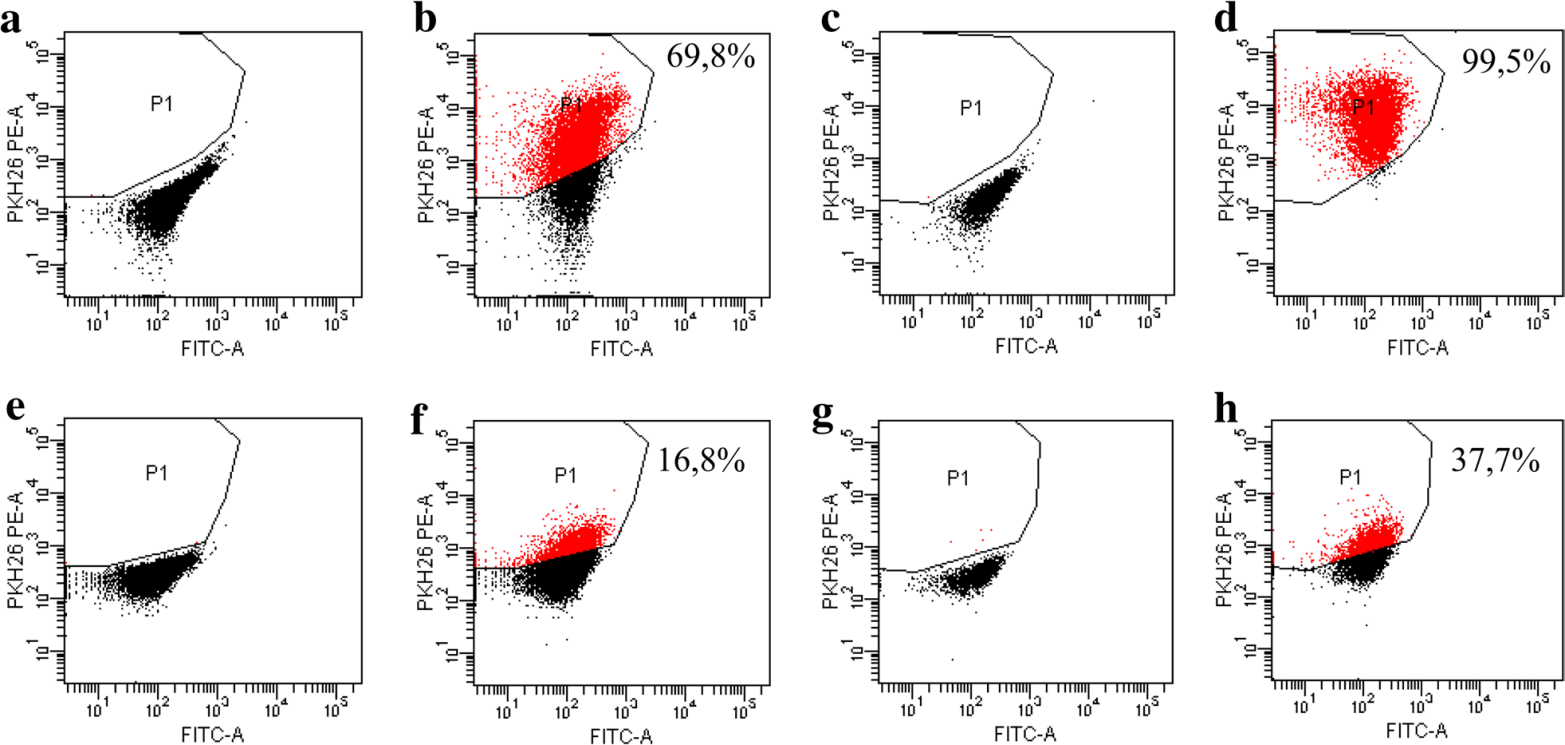
Binding to and engulfment of TMV by GC1415 cells. FACS analysis of GC1415 cells after incubation in the medium (**a**, **c**, **e**, **g**) or with TMV. GC1415 cells were pre-treated with PKH26-labelled TMV for 2 h (**b**, **f**) or 24 h (**d**, **h**). The same samples were treated with crystal violet solution (**e**, **f**, **g**, **h**) as described in “Methods”. Gate P1 was set according to autofluorescence of GC1415 cells incubated in the medium without TMV. The percentage of TMV containing cells is shown

To test the ability of TMV to interact with autologous (parental) cancer cells, GC1415 cells were incubated with PKH26-labelled TMV. FACS analysis indicated that after 2 h of culture about 70 % of GC1415 cells were fluorescent, which increased to 99 % after 24 h (Fig. 3b, d). To discriminate, whether TMV only adhered to the cell surface or were engulfed by the cells, crystal violet was added. After quenching, the percentage of positive cells decreased to 16.8 and 37.7 % at 2 and 24 h, respectively (Fig. 3f, h), thus indicating intracellular localization of TMV.

**Fig. 4:**
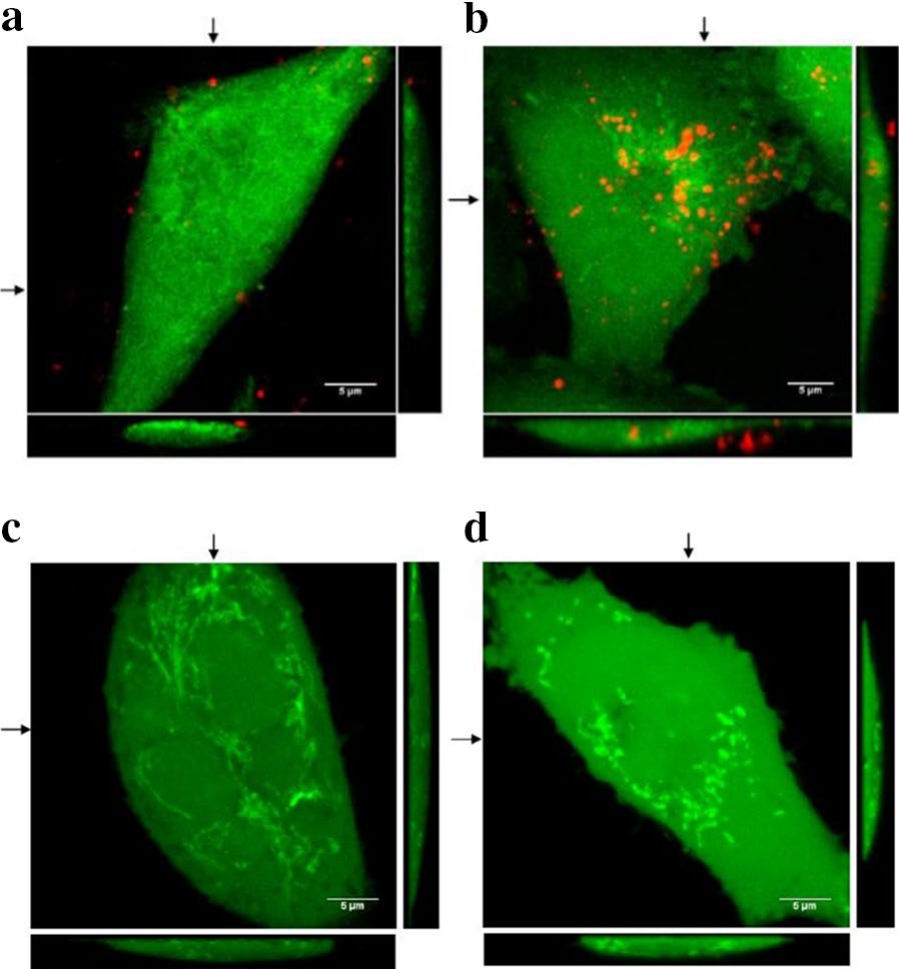
Localization of PKH26-labelled TMV (*red* fluorescence) in GC1415 cells (*green* fluorescence). Maximum intensity projection of three middle confocal planes are displayed. Confocal microscopy images of GC1415 cells exposed to TMV for 2 h (**a**) or 24 h (**b**) demonstrating plasma membrane attached (**a**) and internalised (**b**) TMV; TMV free control for PKH26 labelling for 2 h (**c**) and 24 h (**d**) are shown. A horizontal and vertical sections of the 3D images are also presented. *Arrows* indicates positions of each section

**Fig. 5:**
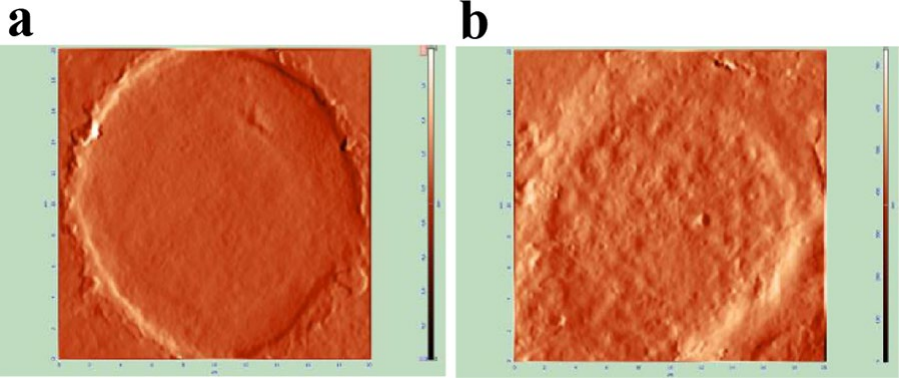
AFM images (phase contrast mode) of GC1415 cells surface without contact (**a**) or exposed (**b**) to TMV for 24 h. Scan area 20 × 20 µm. Phase contrast presentation refers to the phase shift of signal due to the mechanical properties of the sample

To confirm localization of TMV in GC1415 cells confocal microscopy was applied. When GC1415 cells were incubated with PKH26-labelled TMV for 2 h the red fluorescence of the vesicles was associated with plasma membranes (Fig. 4a), however following a 24 h incubation the majority of TMVs were detected inside the cells (Fig. 4b). TMV localized intracellularly were of different size. AFM analysis of control and TMV-treated GC1415 cells showed at 24 h, but not 2 h, significant differences in their surface outlook. The cells not exposed to TMV (Fig. 5a) showed smooth surface and sharp contour. After incubation with TMV (Fig. 5b) the cell surface was rough, uneven with visible protuberations. The cells edges were thicker and diffused of torus shape.

### Tumour growth and angiogenesis

**Fig. 6:**
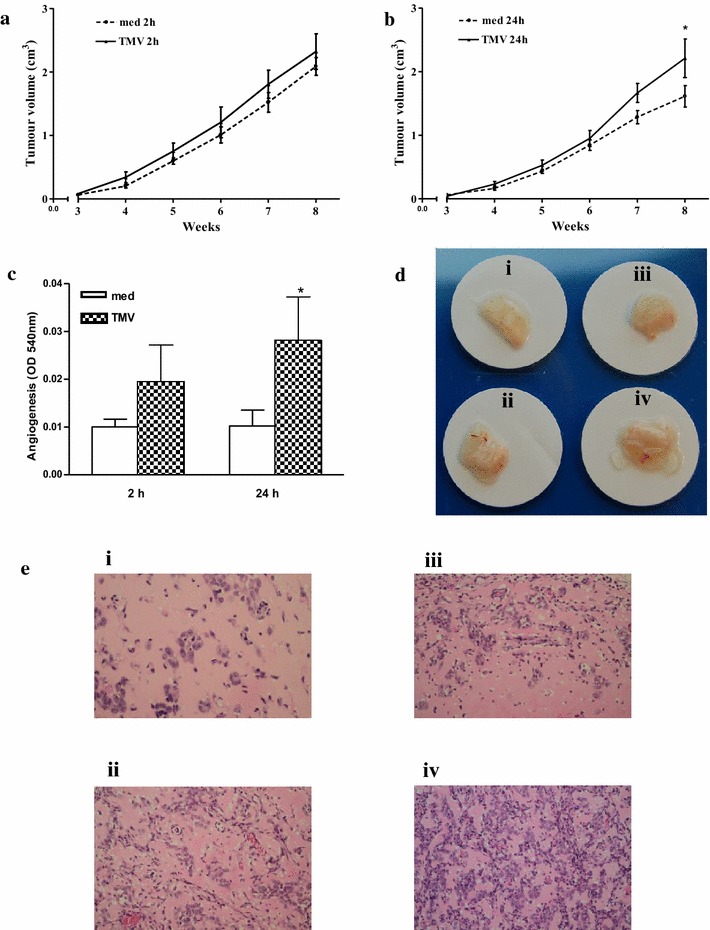
TMV effect on the GC1415 tumour growth and angiogenesis in NOD SCID mice. Before engrafting into NOD SCID mice GC1415 cells were or not exposed to TMV in vitro. Kinetic curve of tumour growth of cells exposed (or not) to TMV for 2 h (**a**) and 24 h (**b**). Angiogenesis as assessed quantitatively by haemoglobin content (**c**). *Bars* represent the mean ± SD from three experiments. Angiogenesis visualised as Matrigel plugs photos (**d**) and histological analysis (**e**). *i*-cells alone, *ii*-cells + TMV after 2 h incubation, *iii*- cells alone and *iv*- cells + TMV after 24 h pre-exposure. *Significantly different (*p* < 0.01) in comparison to control

Inoculation of NOD SCID mice with GC1415 cells led to the formation of tumours in all animals. Palpable tumours appeared around 3 weeks with a rapid growth thereafter. Observation period was 8 weeks. No metastases in the lung, liver, spleen, kidney, lymph nodes were detected either macro- or microscopically. Engraftment of GC1415 cells exposed to TMV in vitro for 2 or 24 h led in the latter case in a slight, but significant, increase of tumour volume as compared to untreated cells (Fig. 6a, b). Metastases were also not detected. Angiogenesis measured quantitatively by haemoglobin content showed significant increase when GC1415 cells were pre-exposed to TMV for 24 h, but not 2 h (Fig. 6c). Visualization of excised Matrigel plugs also showed the same (Fig. 6d). Histological analysis of the same plugs revealed that when tumour cells were used alone, examined areas show either no or few vessels (Fig. 6e, i, ii). When Matrigel Matrix containing GC1415cells and TMV were implanted, both vessels and cells were more numerous, less when tumour cells were pre-exposed to TMV for 2 h (Fig. 6e, ii) and significantly more pronounced in case of cells preincubated with TMV for 24 h (Fig. 6e, iv). It was concluded, that TMV may enhanced tumour growth and angiogenesis.

## Discussion

The role of MV and TMV in cancer is unclear. They are present in the circulation [5, 23] and probably involved in cancer progression, as shown by association of MV (not necessarily of tumour origin) level with poor survival of cancer patients [4]. They may act by inhibiting/hiding antitumour immune response [10] or like tumour-derived exosomes by interacting directly with tumour cells [7, 13, 24]. Current data clearly indicate that tumour microenvironment plays a major role in the inhibition or enhancement of cancer progression. TMV that are present at the tumour site [1] may either induce cellular antitumour response [25] and release antitumour soluble factors [26] or, more likely, exhibit a tumour supporting role [27]. The latter may be due to the induction of growth factors production by tumour cells themselves or infiltrating cells, including macrophages, and/or by promoting the differentiation of myeloid cells into immunosuppressive cell subsets [28].

We have previously reported increased levels of circulating MV and their characteristics in blood of gastric cancer patients [5]. Since it is difficult to determine precisely which of these MV are of tumour origin, this gastric cancer cell line was established and its TMV were isolated and characterized in order to compare them with circulating MV. The immunophenotype analysis confirmed the other data showing that the expression of some determinants on TMV is much lower than on cells [26, 29, 30]. Only CCR6 and CD44v6 expression was comparable on both, cells and their TMV. CCR6 receptor was detected on some cells of pancreatic, colorectal and lung cancer cell lines and their TMV [26], circulating tumour cells [31] and plasma MV of gastric cancer patients [5]. This may suggest its involvement in cancer progression [32]. CCR6 is being identified as a potential new prognostic marker in some types of tumours and the axis of CCL20/CCR6/IL-17 may be a new therapeutic target in cancer [33]. The high expression of CCL20 may contribute to the selective recruitment of CCR6 positive cancer cells and metastases formation [32]. We have previously demonstrated that CCR6 transferred via TMV to monocytes is biologically active, as monocytes acquired chemotactic activity to CCL20 [26]. Based on that, it is reasonable to assume that CCR6 transfer between cancer cells, as exemplified by the attachment of PKH26-labelled TMV, may be a likely mechanism responsible for the enhancement of tumour growth. The presence of CD44H, CD44v6 (hyaluronian receptors) on TMV may also facilitate their interaction with cancer cells, known to abundantly express hyaluronian [34]. This may be analogous to its role in interaction of cancer cells with monocytes, resulting in the induction of reactive oxygen intermediates production [22]. Also, high expression of CD44v6 determinants on TMV may suggest their role in tumour progression, as soluble matrix delivered by CD44v6 is required for premetastatic niche formation [35]. Taken together, it is suggested that CD44H, CD44v6 and CCR6 may be involved in attachment rather than internalization of TMV. By analogy to exosomes, internalization of TMV may occur by various pathways of uptake [7], because the cleavage of exosome surface proteins reduced their association with target cells [12].

The expression of tumour markers (Mucin1, EMMPRIN, EpCAM, EGFR) on TMV was low or absent, despite their presence on cells. Although almost all cells were HER-2/neu positive, small proportion of TMV expressed this marker. The presence of mRNA for HER-2/neu, MAGE-1, -2 in TMV was also detected, although it was lower than in the tumour cells. These mRNAs were also found in circulating MV in plasma of gastric cancer patients [5]. The mRNA for interleukin-8 [8], EGFRvIII [36] and for housekeeping genes such as ACTB, GAPDH [24] were detected in the cargo of TMV and tumour-derived exosomes and shown to be transferred to monocytes [8], brain microvascular endothelial cells [36] and tumour cells [24], respectively. Moreover, TMV containing EGFRvIII released by more aggressive glioma cells were found to be responsible for the transfer of this receptor to indolent cells, which in turn caused the activation of transforming signaling pathways (MAPK and AKT) and changes in other gene expression (VEGF, Bcl-xL, p27) resulting in morphological transformation [37]. Altogether, it is reasonable to assume that the transfer of HER-2/neu molecule, its mRNA as cargo carried by engulfed TMV, and in result HER-2/neu expression by cancer cells may be associated with tumour progression, as observed in breast cancer [38].

The size range of TMV between app. 60–900 nm and the most frequent particle sizes at 80 nm determined by DLS were comparable to 70–520 and 120 nm by NTA, respectively. Similar size ranges were reported for circulating MV in patients with ovarian and gastric cancers [5, 39]. TMV size determined by AFM was also similar. The smaller values of the height in AFM can be explained as a result of probing soft material by relatively hard cantilevers. Thus, the measured altitudes provide only a comparison of the relative size of objects. Morphological characteristics of TMV by TEM and AFM indicated no major differences in comparison to circulating patients’ MV [5]. Such analysis revealed that both MV and TMV particles are mostly spheroid in shape and of different sizes. TMV possessed high (negative) electrokinetic potential. This is in keeping with higher absolute zeta potential values of MV isolated from plasma of gastric cancer patients, as compared to those from donors’ plasma [5] and highly metastatic sublines of rat prostate cancer cells [40]. Although we have no formal proof, similar characteristics (size, morphology, electrokinetic potential) of TMV isolated from GC1415 cell line may support our previous findings that at least some of MV found in blood of gastric cancer patients are of tumour origin [5]. Yet, their role in this type of cancer has to be established.

The present data from FACS, AFM and confocal microscopy provided unequivocal evidence that TMV attached to and were internalized by autologous tumour cells. Both FACS and confocal microscopy indicated that at 2 h of exposure the majority of TMV were seen extracellularly, while at 24 h a large proportion of TMV was present inside the cells. Thus, between 2 and 24 h the entry of TMV occurred. TMV localized intracellularly were of different sizes, implicating their indiscriminate entry to the cells. This suggests that not only exosomes [9] may be taken up by cancer cells. There is an open question whether once engulfed TMV may be transfered to other cells. Furthermore, AFM analysis revealed that the exposure of cancer cells to TMV for 24 h, but not 2 h, induced noticeable structural changes in the cells. TMV-untreated cells had smooth surface and sharp edge, as demonstrable by rapidly changing height at this point during examination. In contrast, the cells exposed to TMV showed blurred edge. This can be explained as caused by possible stronger adherence of TMV-treated cells to the slide and may be compatible with the possible alterations in cell movement. The question arises whether similar situation occurs at the tumour site where TMV are also present [1]. The cell surface showed crumb-like roughness and some protuberations, indirectly indicating intracellular localization of TMV. The present findings are in keeping with the data on uptake of autologous exosomes by ovarian cancer cells [7]. Although, as judged by size, exosomes may be present among total TMV studied, they constitute only a part of all TMV. As PKH26 labels all TMV, it may suggest the entry to the cells both larger and smaller particles.

Altogether, the above data suggest the important role of TMV in communication between cancer cells, which may lead to changes of their biological behaviour. In fact, in vitro exposure of GC1415 cells to TMV, for 24 h, but not 2 h, enhanced tumour growth and angiogenic activity of tumour cells in NOD SCID mice. This is consistent with time of attachment to tumour cell surface (2 h) and engulfment of TMV (24 h), indicating that for these effects intracellular, rather than extracellular, localization is important. It is of interest that enhancement of tumour cells induced angiogenesis was associated with an increased number of cancer cells in Matrigel plugs, This may imply the important role of TMV in supporting tumour growth. It is debatable whether enhancement of tumour growth is connected with transfer of HER-2/neu mRNA and some TMV determinants, e.g. CD44H, CD44v6, CCR6, which are known to facilitate interaction of TMV with monocytes [8, 41]. There may be a special role for HER2 (EGFR) as molecule associated with CD24 which together with phosphorylated Akt promotes breast cancer cell survival [42]. The exact mechanism of tumour growth enhancement by TMV may be multifactorial and requires further studies (on the way).

## Conclusions

TMV obtained from GC1415 cells showed significant heterogeneity in immunophenotype, size and morphology. TMV expressed some tumour markers and several molecules which may facilitate their attachment to parental cancer cells. Uptake of TMV, carrying these molecules and their mRNA, by GC1415 cells resulted in enhancement of tumour growth and angiogenesis in NOD SCID mice. The presented data support hypothesis of autocrine tumour loops [43] in which TMV serve as macro-messengers in delivering signals and molecular cargo to the neighbour tumour cells. Therefore, the engulfment of TMV by cancer cells may qualify them as potential natural carriers of anticancer agents.

## Abbreviations

TMV: tumour-derived microvesicles
mAbs: monoclonal antibodies
MV: micro(nano)vesicles
NTA: nanoparticle tracking analysis
PE: phycoerythrin
FITC: fluorescein
APC: allophycocyanin
PBS: phosphate buffered saline
FACS: fluorescence activated cell sorting
DLS: dynamic light scattering
TEM: transmission electron microscopy
AFM: atomic force microscopy
